# Detention of children and adolescents under mental health legislation: a scoping review of prevalence, risk factors, and legal frameworks

**DOI:** 10.1186/s12887-023-04464-6

**Published:** 2024-01-04

**Authors:** Lisa Schölin, Zack Tucker, Arun Chopra, Rohan Borschmann, Colin McKay

**Affiliations:** 1 Centre for Pesticide Suicide Prevention, University of Edinburgh, Edinburgh, UK; 2 Masters student at University of Edinburgh, Edinburgh, UK; 3 Mental Welfare Commission for Scotland, Edinburgh, UK; 4 Centre for Mental Health, Melbourne School of Population and Global Health, The University of Melbourne, Melbourne, Australia; 5 Centre for Adolescent Health, Murdoch Children’s Research Institute, Royal Children’s Hospital, Melbourne, Australia; 6 Department of Psychiatry, Warneford Hospital, University of Oxford, Oxford, OX3 7JX UK; 7 Melbourne School of Psychological Sciences, The University of Melbourne, Melbourne, Australia; 8 Centre for Mental Health and Capacity Law, Edinburgh Napier University, Edinburgh, UK

**Keywords:** Compulsion, Involuntary hospitalisation, Mental health detention, Mental health legislation, Children, Adolescents

## Abstract

**Background:**

For individuals with severe mental illness, involuntary assessment and/or treatment (hereafter detention) can be a necessary intervention to support recovery and may even be lifesaving. Despite this, little is known about how often these interventions are used for children and adolescents.

**Methods:**

This global scoping review set out to: (1) map the current evidence around mental health detentions of children and adolescents (< 18 years); (2) identify the clinical, sociodemographic, and behavioural factors associated with detention; and (3) document the views of professionals and young people on the implementation of mental health legislation.

**Results:**

After searching databases of peer-reviewed literature and citation chaining, 42 articles from 15 jurisdictions were included. About one fifth of psychiatric admissions in national register data were detentions, however trends were only available for a few high-income Western countries. The circumstances justifying detention and the criteria authorising detention varied between studies, with a mix of clinical factors and observed behaviours reported as the reason(s) warranting/precipitating a detention. Particular groups were more likely to experience detention, such as children and adolescents from minority ethnic communities and those with a documented history of abuse. There was a notable absence of qualitative research exploring the views of professionals or children and adolescents on detention.

**Conclusion:**

Further research is needed to explore the impact of detention on those aged < 18 years, including national register-based studies and qualitative studies. This is particularly relevant in nations currently undergoing legislative reform.

**Supplementary Information:**

The online version contains supplementary material available at 10.1186/s12887-023-04464-6.

## Introduction

Detaining an individual against their will for assessment and/or treatment for mental illness (hereafter ‘detention’) raises important human rights concerns [1–3]. Detention under mental health legislation requires certain criteria need to be fulfilled, which vary between countries [4]. For children and adolescents this is even more complex, as parents may be able to consent to detention on behalf of the child, depending on factors which may include the child’s age or level of understanding. This could result in children being effectively detained without this being recorded as a mental health detention. In recent years, the use of detention has received increased attention from clinicians, policy makers, and academics due to legislative reform. In the UK, the Mental Health Act in England and Wales is undergoing reform, with conclusions of extensive consultation published in 2018 [5] and a draft Mental Health Bill published in 2022 [6]. In Scotland, a final report of the review of mental health and incapacity legislation was published in 2022 [7]. Reform of frameworks outlining criteria for detention is important, as the number of detentions has increased over time [8] within a wider context of increased burden of disease from mental and substance use disorders globally [9].

Domestic legislation is best viewed in the light of international human rights instruments. The UN Convention on the Rights of the Child (UNCRC) [10] and the UN Convention on the Rights of Persons with Disabilities (CRPD) [11] require strong justification for detention, which should take account of the Convention principles. This includes respect for the evolving capacities of children, that detention cannot be arbitrary, must be objectively justified, and that the basis of detention should be non-discriminatory. The UN Special Rapporteur on the Right of Everyone to the Enjoyment of the Highest Attainable Standard of Physical and Mental Health issued a report in 2017 calling for an end to the institutionalisation of children, and to “take targeted, concrete measures to radically reduce medical coercion and facilitate the move towards an end to all forced psychiatric treatment and confinement” (p.21) [12]. At the very least, any evidence of increased detention and involuntary treatment of children would need extremely strong justification, in the light of this demand. The UN Committee on the Rights of Persons with Disabilities has repeatedly stated that it views current forms of detention as breaching the CRPD, stating that “involuntary commitment of persons with disabilities on health-care grounds contradicts the absolute ban on deprivation of liberty on the basis of impairment (art. 14 [1] (b))” and “the principle of free and informed consent of the person concerned for health care (art. 25)” [13].

In adults, a global systematic review found that the risk of being detained is more than double for those with experience of previous episodes of detention and those with a psychotic disorder [14]. Similarly, a separate review found that individuals from ethnic minority groups are at increased risk of mental health detention [15], compared to their white counterparts, which raises questions on how mental health care can adequately support individuals from all ethnic backgrounds, without discrimination. A systematic review which assessed factors associated with detention of children and adolescents found that also in this group psychotic disorder was associated with higher odds of detention [16]. Other factors included substance misuse, having an intellectual disability, being at risk of harming oneself or others, and being older than 12 years of age. This systematic review also found differences between ethnic groups, but this was only true for Black adolescents compared to their white counterparts. The authors however noted that only a small number of included studies reported on ethnicity [16].

While a previous review have addressed factors influencing the likelihood of children and adolescents of being detained, no review to date has synthesised how legislation is used and any views from children and adolescents themselves, or relevant practitioners, on detention of these age groups.

We undertook a scoping review of the evidence relating to mental health detentions of children and adolescents (aged < 18 years). We aimed to examine/document:

- the incidence of detentions in relation to overall admissions, including changes in detention rates over time;
- the clinical, sociodemographic, and behavioural factors associated with detention (compared to voluntary admission/treatment);
- the views of professionals regarding detaining children and adolescents under mental health legislation; and
- the views of children or adolescents on being detained.

## Methods

The review followed the guidance on scoping reviews set out by the Joanna Briggs Institute (JBI) [9], and the Preferred Reporting Items for Systematic reviews and Meta-Analyses extension for Scoping Reviews (PRISMA-ScR) checklist [17]. No protocol was registered.

### Inclusion and exclusion criteria

Primary research studies published in peer-reviewed journals in English language that included children and adolescents aged < 18 years (up until, but not including, their 18th birthday), health professionals, parents, or carers were eligible for inclusion. It is worth noting that definitions of adolescence is an ongoing debate [18], however this review focused on < 18 years as the UNCRC definition of a child. Qualitative, quantitative, or mixed methods study designs were included, regardless of the type of order (emergency, short-term etc.). Any study of psychiatric care of children and adolescents where compulsory care was part of the study, and for which data could be extracted, was eligible for inclusion. Systematic reviews, conference abstracts, editorials, book chapters and sources published in languages other than English were excluded. Studies involving detention of adults or studies including adults, and children and adolescents where results for those aged < 18 years could not be distinguished from those of adults, and studies that exclusively focused on voluntary psychiatric care, were not eligible for inclusion. Detentions in the criminal justice system, detentions relating to immigration status, detentions within general child protection law, and placements in educational institutions for children with special needs education were also excluded.

### Search strategy

We searched PsycINFO, MEDLINE, and Embase for peer-reviewed journal articles published from database inception to 21 September 2022 that included empirical data and were published in English. We used a combination of search terms related to children and adolescents, mental illness and treatment, mental health legislation, and detention (Supplementary Table 1). The search strategy was developed with assistance from a subject librarian at The University of Edinburgh. Database searches were undertaken by ZT, and titles and abstracts were screened by ZT and LS using Rayyan [14]. Any disagreements were resolved through discussion. Following title and abstract screening, full text screening was undertaken by ZT and checked by LS. Forward citation searches and manual reference searches were undertaken by LS.

### Data extraction and synthesis

Data extraction was performed by ZT and LS and LS subsequently summarised the findings and drafted the manuscript. The key characteristics of studies were extracted into an excel spreadsheet using a combination of a pre-determined proforma (country, data collection period, study design, sample, patient group, and key findings) and iterative extracting additional information (e.g. predictors and type of detention). Criteria for detention, although not an original aim of the review, was extracted from the paper or, where unavailable, from papers that described the legal criteria in that country and denoted as ‘not defined’ where no information could be found. Once we started extracting data we decided to extract information about the type of detention as well, and add it to the original focus of the review, as we deemed it relevant to comment on how studies were not necessarily reporting on the same type of detention. This was relevant to studies that described proportion of detentions, rates, and experience of detention. Descriptive statistics were calculated for quantitative studies that reported on proportion of detentions to all psychiatric admissions and all key findings were summarised using a thematic approach.

## Results

### Study characteristics

**Fig. 1:**
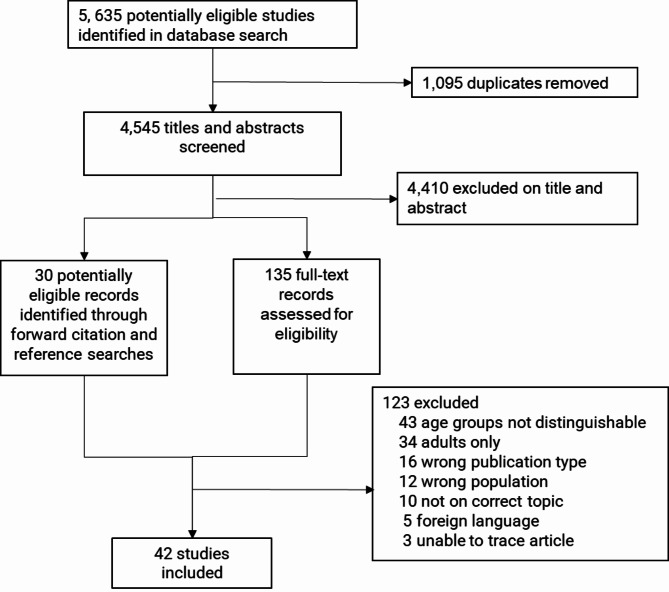
Study selection flow chart

Following title and abstract screening and citation linking a total of 165 full text articles were reviewed resulting in 42 articles included in the review (Fig. 1). Notably, none of the articles excluded at full-text due to foreign language or being unavailable were from countries outside Europe or the USA. Most of the included studies were quantitative (n = 39; 93%) and three were qualitative. Study characteristics are presented in Table 1. Notably, 18/39 studies were from Scandinavian countries (13 from Finland) [19–36] with the remaining studies from other European countries [37–49], North America [50–57], Israel [58], China [59], and New Zealand [60].

**Table 1:** Study characteristics

Reference, country	Period	Study design	Sample	Patient group	Key findings
Ayton et al. (2008) [37], England	2003-06	Cohort study	All (N = 50) children and adolescents admitted to a specialist hospital in England during the study period	Eating disorder	32% of all patients were detained. Significantly more detained patients had depression at admission, reported self-harm or suicidal behaviour, and had more than one hospital admission in the past. Detained patients had younger age of onset of eating disorder, been ill for longer, higher weight (for height ratio) due to being transferred from another hospital following treatment to restore weight, and more required Naso gastric feeding. Overall, detained patients had better outcomes upon discharge.
Chaplin et al. (2015) [38], England	Not stated	Cohort study	151 children and adolescents (6–17 years) admitted to general adult mental health units and intellectual disability specialist units in England.	Intellectual disability	17% of patients were detained. A higher proportion of patients with intellectual disability (21%) were detained compared to those without an intellectual disability (16%) (not significant).
Christy et al. (2006) [50], USA	2000-03	Cohort study	36,551 children and young (2–17 years) who were examined at any Baker Act Receiving Facilities in Florida.	All patients	Mean age was 14.4 years; 48.3% were girls and 47.7% boys; 62.6% were Caucasian, 20.0% Black/African-American, 6.6% Hispanic and 0.3% Asian. Most examinations were initiated due to harm (85.9%), followed by 7.3% for self-neglect and 4.1% for both neglect and harm. Perceived risk of harm was primarily towards self (48.9%).
Clausen et al. (2018) [34], Denmark	2000-13	Cohort study	All 1,953 patients with anorexia nervosa aged 10–17 years admitted for treatment in Denmark (all ages N = 5,767)	Eating disorder	Of all patients aged 10–17 years, 36.2% had an episode of involuntary treatment (admission or detention). Of those who were detained, 90.4% of patients aged 10–14 years and 71.7% of patients age 15–17 years had a registered current registered eating disorder. Analyses of predictors did not stratify by age groups.
Corrigall & Bhugra (2010) [39], England	2001-10	Cohort study	435 adolescents (12–17 years) admitted to an adolescent inpatient psychiatric service in south London	All patients	Mean age of patients was 16.3 years; 49% were Black, 32% White, 3% Asian, and 15% other ethnicity; 53% were girls. Overall, 36% of the study sample had been subject to the Mental Health Act at some point during the admission. Black adolescents with psychosis had higher odds of being detained (OR = 3.0, 95% CI: 1.3–6.7) and other ethnicities (OR = 3.1, 95% CI: 3.1–1.1), compared to their white counterparts.
Deolmi et al. (2021) [48], Italy	2013-15	Cohort study	51 adolescents admitted to Parma Local Health Unit general psychiatric wards	All patients	A total of 21.6% (11 patients) were detained, which varied across the years. The highest number of cases (n = 6) were for conduct disorder, though no statistical analysis was conducted comparing detained patients to voluntary patients.
Ellila et al. (2008) [19], Finland	2000	Cross-sectional	278 children and adolescents (12–17 years) admitted to psychiatric inpatient wards in Finland on a given day in January	All patients	29.5% patients were detained. The largest age group of detained patients was 16–17 years (48%) and 59% were girls. Significantly more detained patients had psychotic disorder (62% vs. 15%, *p* < 0.001) and substance use disorder (9% vs. 1%, *p* = 0.005) than voluntarily admitted patients.
Eswaravel and O’Brien (2018) [40], England	2011-16	Cohort study	85 children and adolescents under the age of 18 years to a mental health trust in south-west London	All patients	Mean age was 15.7 years; 60% were female; 78.7% White, 8.2% were Black, 8.2% Asian and 4.6% Mixed ethnic groups; 16.5% had more than one admission over the period. The most common reason for detention was attempted suicide or deliberate self-harm (56.7%), 62.4% had a recorded history of self-harm, 64.4% were already known to CAMHS. Of those admitted and assessed, most were discharged home (67.3%) while 20.2% were detained under s.2 or s.3 of the Mental Health Act and 12.5% were voluntarily admitted.
Geng et al. (2020) [59], China	2019	Cohort study	196 adolescents discharged from 41 tertiary psychiatric hospitals in 29 provinces of mainland China between 19–31 March 2019	All patients	32.1% were detentions. Detained patients were slightly older, had significantly lower global assessment of functioning (GAF) scores, admitted with psychotic symptoms or aggressive behaviour but were less likely to have depressive symptoms. There was no significant difference in suicidal or self-harming behaviour during the admission. Detained patients had longer length of stay and were more likely to be diagnosed with schizophrenia but less likely to be diagnosed with depressive disorder.
Greenham and Persi (2013) [55], Canada	2009-10	Cross-sectional	Information about admissions received from 25 out of 27 inpatient services in Ontario, Canada.	NA	Detention for psychiatric assessment was reported by 21 units, on average 40% were involuntary (ranging from 0–80%). Admission for treatment was reported by 15 units, on average 5% were involuntary (range: 0–40%).
Hanssen-Bauer et al. (2011) [32], Norway	2005	Cohort study	192 children and adolescents (10–18 years) with admitted to 4 units in Norway (out of a total of 16 units) with a first episode of care starting in 2005. Only included patients admitted within 7 days of referral	All patients	Admission status for compulsory vs. voluntary status was only relevant for patients aged ≥ 16 years^1^. Of these 33.3% were involuntary, ranging from 7–67% between units (*p* < 0.001). Those detained had a higher Health of the Nation Outcome Scales for Children and Adolescents (HoNOSCA) score (mean = 21.0, SD = 6.1 vs. mean = 16.8, SD = 6.3, *p* < 0.001).
Jaworowski & Zabow (1995) [58], Israel	1991-92	Cohort study	78 children and adolescents (< 18 years) admitted for psychiatric care in Beersheva, Israel	All patients	18% of patients were detained. The most common diagnoses related to detention were conduct disorder (38%) and personality disorder (31%), of which the majority were borderline personality disorder (62.5%).
Jendreyshak et al. (2013) [46], Germany	2004-09	Cohort study	10,547 minors (< 18 years) treated in inpatient psychiatric services across 27 administrative districts in Germany.	All patients	29.2% of admissions were involuntary, most of whom were aged 14–15 years (39.7%) and 15–16 (37.3%). The proportion of involuntary admissions decreased over time, from 32.4% in 2004 to 25.7% in 2009, while the length of stay of detentions increased. The highest odds for being detained was for suffering from “mental retardation” (OR = 15.74), other predictors included being adolescent, having substance abuse problems, psychotic disorders, and an admission during duty hours (odds ratios > 3).
Kaltiala-Heino (2010) [20], Finland	2004-06	Cohort study	187 adolescent aged 11–17 years admitted to Tampere University Hospital in Southern Finland	All patients	49.7% of young people were involuntarily referred and 22.5% were involuntarily treated. Mean age was 15 years, 64.2% were girls, 17.1% lived in child welfare institutions and 5.7% lived in foster care. Those admitted involuntarily were more likely to be referred from primary care, from non-psychiatric specialties to lesser extent had received community-based treatment in the past than those admitted voluntarily. Patients who were both involuntarily referred and treated were more likely to have psychotic symptoms, temper tantrums, and were breaking property compared to voluntary patients. Those involuntarily referred were more likely to have violent behaviour, but this was not the case for involuntarily treated. Involuntarily treated, but not referred, were less likely to have depression compared to voluntarily treated patients.
Kaltiala-Heino and Frojd (2007) [21], Finland	2003	Qualitative interview study	44 child and adolescent psychiatrists and psychiatrists in training across 8/21 child and adolescent psychiatric departments in Finland	NA	Psychiatrists did not believe that difficulties in defining severity of mental disorder could be justified using ICD or DSM diagnoses. Acute severity (deemed as presenting as a risk of harm to self or others or loss of life) was differentiated from chronic (leading to regression or impact on development. The criteria for severe mental disorder must be justified alongside the risk that the minor deteriorates unless committed involuntarily, a risk of harm to self or others, and that voluntary treatment is inadequate.
Kaltiala-Heino et al. (2004) [22], Finland	1996–2000	Cohort study	Involuntary admissions of children aged 12–17 in all healthcare settings providing inpatient treatment in Finland	All patients	4.8% of admissions in children (< 12 years) and 22% of adolescent admissions (12–17 years) were involuntary. There was no difference between girls and boys. Involuntary admissions were more likely to be for substance use-related disorders, schizophrenia spectrum disorders, or mood disorders. Involuntary psychiatric admissions increased over time from 2.4 per 100,000 in 1995 to 7.2 in 2000.
Khenessi et al. (2004) [23], Finland	1994–2002	Cohort study	106 adolescents (13–18 years) sent for involuntary treatment at an adolescent psychiatric unit in south-west Finland	All patients	88% were admitted for psychiatric observation while 11% were released from involuntary treatment at admission. Among patients who were admitted for involuntary treatment after observation, more had psychotic symptoms compared to those who were released (41.0% vs. 19.4%, *p* = 0.016).
Kilgus et al. (1995) [51], USA	1988	Cohort study	All 352 psychiatric inpatient admissions (12–18 years) to a state hospital facility in South Carolina, US	All patients	Most admissions were White (71%) and the remaining 29% were African American. Mean age of all admissions was 15.4 years and 55.4% were male. Overall, 78.1% of admissions were detentions, which was significantly higher among African Americans (87.1%) than Whites (74.5%) (*p* = 0.01) with the odds of detention among African American being 2.05 (*p* = 0.043).
Kronström et al. (2016) [24], Finland	2000,2011	Cross-sectional	916 children and adolescents (< 1 years) admitted on a given day to 64/69 wards (2000) and 74/79 inpatient psychiatric wards in Finland.	All patients	Data also reported on in [19]. The proportion of admissions that were involuntary was 18% in 2000 and 19% in 2011. The proportion among admissions in children’s wards was 1% in 2000 and 4% in 2011 while the proportion in adolescent wards was 34% in 2000 and 31% in 2011. None of these differences were statistically significant. Due to the aim of the study, no exploration of characteristics were made within this group of patients.
Kronström et al. (2021) [31], Finland	2000, 2011, 2018	Cross-sectional	1276 inpatients in Finland (93–95% response rate)	All patients	Data also reported on in [24]. The proportion of detained patients remained stable - in 2000 a total of 18% were detained, in 2011 19% and in 2018 22% of patients were detained.
Lindsey et al. (2010) [52], USA	2001-02	Cohort study	1,450 African American minors (< 18 years, total sample also including 18–22 years) presenting at one of the five crisis response centres in Philadelphia, USA.	African American patients	Among those aged under 18 years, 25.6% of those arriving at the psychiatric emergency services (PES) had involuntary status, which was higher among those aged 13–17 years than those 12 years or younger (32.4% vs. 16.3%). The study explored a subsample of minors for which there was an official petition of civil commitment; 445 of these 501 minors were under 18 years. Within this group the commitment decision was involuntary for 59.3%, voluntary for 16.2% while 24.5% had their case dismissed. Subsequent analyses on predictors combined all age groups, including those over 18.
Mears et al. (2003) [41], England and Wales		Cross-sectional	51 of the 76 inpatient CAMHS consultants in England and Wales	NA	63% of respondents had undertaken at least one day’s training in mental health law in the last two years. 37% felt fully up-to-date with law changes relating to children and adolescents and 57% were partially up-to-date. 90% felt that their access to legal advice was at least adequate. 74% either agreed or strongly agreed that guidance on when to use which act was needed, 88% felt that more training on legal issues was needed. Correct responses for criteria for using the Mental Health Act was 68% and 45% for the Children Act. The mean correct response rate around consent relating to children and young people was 77%. Consultants who used the Mental Health Act at least once every six months had significantly higher correct response rate than those who used it less often 93.1 vs. 2.4, *p* < 0.05).
Mears and Worral (2001) [43], England and Wales	2001	Cross-sectional	258 (54% response rate) psychiatrists working in England and Wales	NA	The most common theme was choosing between the Mental Health Act and the Children Act when detaining an adolescent. Other themes related to issues around consent for treatment, social services, and stigma associated with being detained under the Mental Health Act.
Mears et al. (2003) [42], England and Wales	1999	Cross-sectional	663 children and young people (age not clearly stated) inpatients across 71 units in CAMHS units in England and Wales on a given census day	All patients	19% were formally admitted and almost all of these were under sections of the Mental Health Act (n = 119). The proportion of detained patients varied by type of unit (100% in forensic and secure units, 85% in learning disability, 9% in general psychiatry). Detentions were significantly higher among > 16s than < 16s (35% vs. 8%, *p* < 0.01), among boys than girls (23% vs. 16%, *p* < 0.05), among patients with schizophrenia (45% vs. 10%) and personality disorder (16% vs. 3%). Detained patients had significantly higher levels of reported youth had a history of sexual abuse, physical abuse, emotional abuse, multiple self-harming, and requiring one-to-one observation.
Nicholls et al. (1996) [44], England	1983-94	Cohort study	492 young people aged 12–17 years admitted to an inpatient unit for young people in the West Midlands, England	All patients	Among detained patients, 63.6% were male, mode age was 16 years. 6.7% of admissions were detentions at some point during the hospital stay, of these 42.2% were admitted under the Mental Health Act. There was a higher proportion of detentions in later years; 0–9% in 1983-90 compared to 11–27% in 1991-94.
Nyttingenes et al. (2018) [33], Norway	2015	Cross-sectional	96 admitted patients (13–17 years) to 10 (out 16 wards) acute and combined psychiatric wards in Norway.	All patients	18.8% of the sample were detained, which was higher among those aged 16–17 years (22.2%) compared to 13–15 years (12.1%). For voluntary admitted patients, informal pressure from parents was associated with higher perceived coercion whereas for detained patients more informal pressure from their parents was associated with a lower perception of coercion.
Park et al. (2011) [60], New Zealand	2002-07	Cohort study	332 children and adolescents < 18 years admitted to general inpatient psychiatric unit in Hamilton, Auckland	All patients	61.4% were detained (“involved the Mental Health Act”). Significantly higher proportion detained patients were boys (74.2% vs. 49.7%, *p* = 0.000), Maori were (compared to Caucasian youth; 68.2% vs. 57.1%, *p* = 0.04), and due to “deterioration of mental state” (79.7%) or aggression (75.0%). Those admitted under the Mental Health Act had longer length of stay than voluntary admissions (11.23 days vs. 3.75 days).
Pelto-Piri et al. (2016) [35], Sweden	2002-03	Cohort study	142 young people aged 10–18 years across all 16 child and adolescent clinics in Sweden who used coercive care	All patients	Median age was 16 years, 64.1% were girls, 9.2% were asylum seekers. Most common diagnoses were eating disorders, psychosis, depression, and neuropsychiatric disorders. 21.1% also had substance abuse. The most recorded reason for coercive care was the ‘protection’ argument^2^ in 96% of Psychiatric Care Certificates and 99% of complete medical records, followed by ‘treatment requirement’ in 69% of complete medical records and 56% of Psychiatric Care Certificates, and ‘parental support’ on 48% of medical records and 24% of Psychiatric Care Certificates.
Persi et al. (2016) [55], Canada	2007-08	Cohort study	225 discharges of children and adolescents (5–17 years) in 26 acute hospitals in Ontario, Canada.	All patients	80% of admissions were detentions. A higher proportion of detained patients were adolescents (89% vs. 73%, *p* < 0.05), not living with family (27% vs. 4%, *p* < 0.05), and at risk of suicide (89% vs. 71%, *p* < 0.05). Of detained patients considered at risk of suicide at referral, 45% were considered risk of suicide at the psychiatric assessment (*p* < 0.05) compared to 66% of voluntary patients (*p* < 0.05). Length of stay was shorter for detained compared to voluntary patients (Mdn = 6 vs. Mdn = 6, *p* < 0.05). Among those referred on detained status, only 13% remained detained after psychiatric inpatient assessment.
Ramel et al. (2015) [36], Sweden	2011	Cohort study	261 children and adolescents receiving psychiatric care from Child and Adolescent Psychiatry emergency unit in Malmö, Sweden.	Unaccompanied minors	10.7% were detentions, which was significantly higher among unaccompanied refugees than other patients (19.6% vs. 8.3%, *p* = 0.024). There was a higher proportion of detentions of boys (71.4%), and among unaccompanied refugee minors all detentions were of boys compared to 56.2% of accompanied minors.
Rice et al. (2021) [53], USA	2017	Qualitative interview study	25 children and adolescents (13–17 years) admitted for suicidality to a CAMHS unit in a psychiatric hospital in a Southeastern State, USA.	Suicidal patients	The young people felt stigmatised both before and after arriving at the hospital for involuntary inpatient treatment. Many felt disregarded and dehumanised during the admission, leading to increased sense of stigma. Receiving and providing support from other young people in the hospital was an important part of the admission experience to not feel alone. Time away from things like social media, friends and family gave the young people an opportunity to engage with practices to cope with stressors which had positive outcomes such as reduction in stress levels.
Siponen et al. (2007) [25], Finland	1996–2003	Cohort study	9865 admissions of adolescents (12–17 years) to psychiatric hospitals across Finland.	All patients	23.6% were detained, which increased from 16.2% in 1996 to 26.3% in 2003 (a 1.6-fold increase). Between regions the proportion ranged from 4–32% of all admissions. Across all years, the rate of detention was 22.4 per 100,000 (95% CI: 20.94–23.92), which ranged between regions from as low as 5.06 (95% CI: 1.63–15.70) to 36.67 (95% CI: 28.70–46.84) per 100,000. There was a positive correlation between standardised rate of detentions and child welfare placements (correlation coefficient = 0.44, *p* = 0.048).
Siponen et al. (2011) [26], Finland	1996–2003	Cohort study	520 adolescents aged 13–17 in two hospital districts in Finland (above and below average rate of involuntary admission and detention)	All patients	Data also reported on in [25]. In the area with above average rate of involuntary admissions was 8.6 per 1,000 and detention (see Supplementary Table 2 for definition) was 5.8 per 1,000. For the below average area the rate was 3.9 and 1.9 per 1,000, respectively. Overall use of compulsory care differed significantly between the two regions (8.8 vs. 3.9, *p* < 0.0001). The above average district had a significantly higher prevalence of diagnoses of schizophrenia and personality disorders among detained patients (personality disorder was also higher among voluntary patients), significantly lower employment rate, rate of further education, migration to and from the area, higher number of divorces and single parent families, higher youth and overall crime rate, exclusion, individuals in detoxification treatment, patients in A-clinics, and mental health service use. The above average district had more outpatient service positions, adolescent psychiatric positions, more private and public welfare institutions for children, non-institutional welfare support, but significantly less adolescent psychiatry outpatient visits (119.2 per 1,000 below average district vs. 30.2 in above average district, *p* < 0.0001).
Siponen et al. (2012) [27], Finland	1996–2003	Cohort study	9,865 admissions to inpatient psychiatric treatment aged 12–17 years across Finland	All patients	Involuntary treatment increased from 14.4% in 1996 to 21.4% in 2003. Among all involuntary treatments during the study period, coercive measures (“seclusion, restraint, involuntary i.m. medication and physical holding”, p. 1403) were used in 27%. The most common diagnoses for involuntary treatment episodes were mood disorders (28.6%), conduct disorders (26.8%), and schizophrenia group disorders (20.1%). Coercive treatment within those treated involuntarily was higher among girls than boys (29.5% vs. 23.6%, *p* = 0.005) but there was no difference between younger (12–14 years) and older (15–17 years) adolescents.
Smith et al. (2004) [56], Canada	1998–2003	Cohort study	All patients < 16 years (total number not reported) admitted to a paediatric ward to one regional hospital in Ontario, Canada.	Paediatric patients	8.9% of admissions were involuntary; in 1998-99, 0 of the 25 admissions were done using involuntary measure compared to 11 out of 45 (24.4%) in 2002-03. Of the total 15 involuntary admissions across all years, 40% were for suicidal behaviour, 33% for behaviour disturbance, 13.3% for mood disorder, 7% (1 admission) for mood disorder and suicidal behaviour, and 7% (1 admission) for psychosis.
So et al. (2021) [47], Netherlands	2008-17	Cohort study	All 227 emergency admissions of children and young people (6–18 years) following outpatient emergencies in Amsterdam and Greater Rotterdam, the Netherlands	All patients	39.6% of admissions were compulsory. Regression analyses found significant association between being compulsory admitted and prior compulsory emergency admission (OR = 10.48, 95% CI: 2.44–45.09), severe or moderate suicide risk score (OR = 4.10, 95% CI: 1.63–10.30), being a danger to others (OR = 2.82, 95% CI: 1.00-7.96), lack of motivation for treatment (OR = 22.77, 95% CI: 8.48–61.14), lack of compliance with medication (OR = 4.31, 95% CI: 1.75–10.61), and all DSM disorders aside from relational and adjustment disorders (OR = 40.41, 95% CI: 1.12-1,458.79).
Sourander & Turunen (1999) [28], Finland	1990 and 1993	Cohort study	All 1,776 adolescents aged < 18 years discharged from discharged from psychiatric treatment in Finland	All patients	Data also reported on in [29]. 8.3% of patients were treated under compulsory measures in 1990, compared to 6.5% in 1993. The prevalence and incidence of compulsory care per 10,000 population increased from 7.2 to 8.2 (prevalence) and from 4.7 to 5.9 (incidence) from 1990 to 1993. The rate was highest in the 12-17-year group and among boys.
Sourander et al. (1998) [29], Finland	1990 and 1993	Cohort study	All 1,014 children and adolescents aged 12–17 years discharged from psychiatric treatment in Finland	All patients	The number and proportion of compulsory treatment of minors was 65/462 (14%) in 1990 and 62/552 (11%) in 1993. Most (> 90%) were aged 16–17 years, 51.2% were male (57% in 1990 and 45% in 1993), and most were admitted to adult wards (71.1%). The most common diagnosis was for psychotic disorder, which was a significant predictor for involuntary treatment (OR = 4.68, 95% CI: 2.69–6.81). Other predictors included being 15–17 years (OR = 1.48, 95% CI: 1.24–1.76) and being admitted to an adult ward (OR = 3.14, 95% CI: 1.99–4.94). The prevalence of compulsory care ranged from 0 to 3.4 per 10,000 minors. The proportion of compulsory treated minors, of all admissions, ranged from 0–21%.
Stein et al. (1988) [57], Canada	1977-84	Cohort study	All 294 discharged patients (no age provided) from an adolescent psychiatric unit it Ontario, Canada.	Suicidal patients	Among all discharges, 25 patients (8.5%) were detained at some point during the admission of whom 23 could be followed up. Of those detained, 52% were boys, mean age was 16.5 years for boys and 17.0 years for girls. Five (20%) of the detained sample had died by suicide during the follow up period (approximately five years). The diagnoses of those who were detained were (n = 11), personality disorder (n = 5), major affective disorder (n = 4), and other diagnosis (n = 3).
Tolmac & Hodes (2004) [45], England	2001	Cross sectional	113 adolescents (13–17 years) across adolescent psychiatric units and in-patient psychiatric wards in Greater London, focusing on a subsample of 55 adolescent with psychotic disorders	All patients	The mean age among all admitted young people was 16.2 years. Among adolescents with psychotic disorder 70% were male, 45% were White, 35% were Black, 13% were Asian, and 7% were of Other ethnicity. Overall, 62% of adolescents were subject to the Mental Health Act 1983 at some point during admission. There was no significant difference in detention at any point, but Black adolescents were more likely to be subject to the Act upon admission (63% vs. 16% White, *p* < 0.03).
Turunen et al. (2010) [30], Finland	2003	Qualitative interview study	44 child and adolescent psychiatrists and psychiatrists in training across 8 of the 21 child and adolescent psychiatric departments in Finland	NA	Data also reported on in [21]. Psychiatrists in general believed that detaining minors under different criteria to adults was appropriate. Detaining minors, who due to their age may not be able to weigh up the benefits of treatment, was seen as a possible early intervention to prevent future mental ill health and the criteria was seen as appropriately broad for this age group, but possibly too narrow for adults. Lack of definition of severe mental disorder was seen as potentially leading to different application of the law across the country.
Voultous et al. (2020) [49], Greece	2005-14	Cohort study	131 involuntary admitted minors in Thessaloniki, Greece.	All patients	Mean age was 14.19 years and 61% were boys. Of all involuntary admitted minors, 69.7% were discharged to go to their home while the remaining patients primarily were transferred to an institution. Among all patients, 48.9% had behavioural disorder and impulsive behaviour, 21% had pervasive developmental disorder, 14.5% had no diagnosis and 10.7% had intellectual disability. A smaller proportion had schizophrenia (4.6%), drug or alcohol abuse (3.1%) and personality disorder (2.3%). 7.6% of patients were re-hospitalised (follow-up period unclear)

In 36 studies the study samples, or part of an overall sample, were children and adolescents aged 5–17 years, in four the study sample were psychiatrists, and in one study the data related to services. Data were collected over a period ranging from one day to 11 years. In the 39 quantitative studies, 13 covered a national sample, 14 service level, nine regional level, and three city level.

### Legal frameworks for detaining children and adolescents

The legal criteria and types of detentions were mapped out to better understand differences between studies and countries (Supplementary Table 2). Criteria consistently included the presence of mental disorder and risk of harm, with differing qualifiers as to the severity of mental disorder or level of risk. The English and Finnish criteria also included the availability of suitable treatment. Only the criteria cited in the Greek study [49] appear explicitly to include lack of competence to reach a decision about one’s own treatment.

Included studies concerned different frameworks for detention, with some variation between them and a wide range of periods where a child or adolescent was deprived of liberty for assessment or treatment of mental illness (Supplementary Table 2). Twenty studies (47.6%) did not define the detention period, and Philipps et al. [61] noted that there is no limit to the period in which someone can be detained in China. In five studies detention lengths ranged from 24 h in a place of safety (i.e., not an admission to a psychiatric ward) to 72 h [40, 50, 53, 55, 56], while three studies included both shorter periods of detention and longer periods from 14 days up to 6 months [42, 44, 54]. Pelto-Piri et al. [35] focused on analysis of reasons for compulsory care and the period from when a Care Certificate is made to decision on compulsory care is taken (24 h after Care Certificate is created). Studies relating to the Finnish Mental Health Act 1990 [19–27, 30, 31] appeared to refer to any assessment or treatment period.

### Prevalence, incidence, and trends in detention

The proportion of detentions in relation to overall inpatient admissions, where relevant, could be calculated for 30 studies and ranged from 7% in a nation-wide sample in Finland [29] to 80% among discharges of 225 minors in Canada [55]. The mean across all studies was 30% (median = 24%), with a mean of 21% in the eight studies that used nationwide samples (median = 20%). Ten studies reported on changes in detentions over time, most being from Finland where the rate of detention increased from 7.2 to 8.2 per 10,000 population and incidence rate from 2.7 to 5.9 between 1990 and 1993 [28]. Prevalence of detentions was 14% in 1990 and 11% in 1993 [29], while in later years higher prevalence was reported but with little change across the years (18% in 2000, 19% in 2011, and 22% in 2018) [31]. Two additional studies, from Canada [56] and England [44], reported on increases in detentions but both using service-level data making it difficult to comment on whether it was part of an overall trend. Only one study, by Jendreyshak et al. [46], found a decrease in detentions from 32% in 2004 to 26% in 2009 across 27 districts in Germany.

### Factors associated with detention

#### Clinical and behavioural factors

In 22 studies factors associated with detention were explored either univariately or in multivariate models. Compared to children and adolescents who had voluntary status, those detained had a higher prevalence of psychosis, or psychotic symptoms [19, 20, 29, 39, 46, 59], conduct disorder [27, 48, 58], substance use disorder [19, 22, 46], schizophrenia [42, 59], and personality disorder [42, 58]. So et al. [47] found that detention was significantly associated with wider diagnostic categories (‘internalising’, ‘externalising’ and ‘other’) derived from the Diagnostic and Statistical Manual of Mental Disorders (DSM) other than a category for ‘relational and adjustment disorder’. In children and adolescents with an eating disorder, Ayton et al. [37] found a higher prevalence of depression and Jendreyshak et al. [46] found that ‘mental retardation’ was the strongest predictor for detention.

Other, less frequently factors reported included: self-harm and/or suicidal behaviour at admission [37] requiring one-to-one observation [42], deterioration of mental state [60], longer length of stay [60], referral from non-psychiatric specialty [20], temper tantrums and violent behaviour [20], aggressive behaviour [59, 60], learning disability [46], being treated in an adult ward [29], admission during out of hours [46], experience of abuse [42], being an unaccompanied refugee [36], positive correlation between detentions and child welfare placements [25], higher Health of the Nation Outcome Scales for Children and Adolescents (HoNOSCA) score (a general health and social functioning tool) [32], prior emergency admission [47], danger to others [47], lack of motivation [47], and lack of compliance with medication [47].

#### Sociodemographic and socioeconomic factors

Most studies that compared non-detained individuals found no difference in the proportion of detentions by sex [19, 20, 22, 23, 47, 55], two studies found a higher proportion of boys [42, 60], and one study found a significantly higher proportion among girls [46]. In studies that did not compare with non-detained individuals, seven found that detentions were predominantly girls [19, 23, 25, 26, 47, 55, 59], and in three predominantly boys [36, 45, 60]. Three studies reported differences between ethnic groups. Tolmac and Hodes [45] found that Black adolescents were more likely to be detained on admission than their White counterparts, but there was no significant difference in being detained during the admission. In contrast, one study from England found that psychosis patients from Black or ‘Other’ ethnic backgrounds were more likely to be detained during the admission [39], a US study found higher proportion of detentions among African American compared to White patients [51], and a New Zealand study found higher proportion of detentions among Māori compared to Caucasian adolescents [60]. It is noteworthy that, in many studies, ethnicity was not included as a variable. Finally, detained patients were reported to be older than voluntary patients in four studies [29, 33, 42, 46].

Siponen et al. [26] explored wider socioeconomic and service-related factors and their impact on detentions. They found that the areas with above average rate of detentions typically fared poorer on factors related to social environment, such as a higher divorce rate, lower employment rate, and rate of clients in substance misuse treatment. However, there were more outpatient service (staff) positions, adolescent psychiatric positions, more private and public welfare institutions for children, non-institutional welfare support, but fewer adolescent psychiatric outpatient visits in the above average detention area. While this ecological study could not prove causation, the authors noted that there may be an association between socio-economic disadvantages and detentions and “to reduce the use of involuntary care in adolescent psychiatry and child welfare, approaches focusing on the well-being of families may be indicated” (p.660) [26].

### Views on detention of children and adolescents

Five studies included views on detaining children and adolescents; two were cross-sectional surveys of English psychiatrists [41, 43], two were qualitative studies of Finnish psychiatrists [21, 30], and one study included the views of children and adolescents [53].

A survey from England in the early 2000s showed that psychiatrists felt up to date with legislative changes, but the majority agreed that more guidance and training was needed. Importantly, 18% provided an incorrect answer to the question whether parents’ consent to treatment overrides a child’s refusal [48]. A subsequent survey by Mears and Worral [43] found that the main issues for using the Children Act or the Mental Health Act were (i) choosing which Act to use; (ii) general issues around consent for treatment and social services; (iii) stigma; and (iv) conflict between the child’s wishes and parental consent [43]. Studies from Finland were more in-depth and specifically focused on views on the criteria for detention. Kaltiala-Heino and Fröjd [21] interviewed 44 Finnish child and adolescent psychiatrists who all believed that severity of mental disorder could not be arrived at by using ICD or DSM diagnoses (Supplementary Table 2). They differentiated between acute and chronic severity and felt that the criteria for ‘severe mental disorder’ must be justified alongside risk of deterioration, a risk of harm to self or others, and that voluntary treatment is inadequate [21]. Turunen et al. [30] found that arguments supporting a broader criteria for minors included the need for paternalistic intervention due to inability of minors to weigh up need for treatment, detention as an early intervention to prevent future deterioration, and difficulty in diagnosing minors with a mental illness. Argument against a broad criteria for minors was that lack of a definition of severe mental disorder could lead to differences in the interpretation and application of the law, and overall psychiatrists argued that the criteria is too narrow for adults rather than too broad for minors [30].

Finally, Rice and colleagues [53] interviewed 25 children and adolescents staying in a crisis stabilisation unit in USA. Participants felt stigmatised before and after arriving at the hospital for involuntary treatment; some arriving in handcuffs and escorted by police officers leading many to feel disregarded and dehumanised during the detention process. However, receiving and providing peer support was important to feel that they were not alone. By discharge, interviewees reported improvements included ‘opening up’ in group therapy, supporting others, and receiving support from clinical staff. Time away from social media, friends and family gave the young people an opportunity to engage with practices to cope with stressors with positive outcomes including reduced stress levels [53].

## Discussion

About one fifth of psychiatric child and adolescent inpatients are treated while detained under mental health legislation, but evidence on how this might be changing is limited. This review has demonstrated that the evidence base relates to data mainly from a few Western high-income countries (HICs) and great variations in the type of detention order studied. Any deprivation of liberty and treatment against one’s will is important and relevant, but despite many included study indicating a significant proportion of inpatients are treated while detained there is little exploration of patient or practitioner views.

Most included studies were conducted in HICs, primarily in Europe, where financing of mental health services is higher. In 2020, expenditure for mental health services in low- and middle-income countries (LMICs) was lower than in upper-middle and HICs, as were the number of health workers in Child and Adolescent Mental Health Services (CAMHS), and insurance arrangements means patients often pay out of pocket for mental health services and/or psychotropic medicines [62]. Lack of funding for mental health services likely deter individuals to seek care, alongside stigma which has been shown to differ between Eastern (Asian) and Western countries [63]. The current review leaves many questions of how children and adolescents are detained in LMICs when voluntary psychiatric care is no longer an option. Coverage of data on major mental disorders is poor in LMICs [64] and future work should focus on collecting data on involuntary psychiatric care of children and adolescents in countries where this is currently lacking. This also includes research into detention of children and adolescents in HICs, as we note a lack of studies from Australia and the USA. Australian research, such as a recent descriptive study of administrative data relating to CAMHS, did not report on legal status of admissions [65]. The reason for the lack of studies noting the legal status is unclear, as data from the Australian Institute of Health and Welfare breaks down involuntary treatment by age [66]. In the USA, many states do not report on detentions and of those who do, only six states separate data on adult and minors (though this article does not define minor and definitions varies in different states) [67].

There is a notable lack of qualitative research on detention of children and adolescents. The only study we found involving lived experiences included short detention in crisis management settings due to suicidality [53]. While these views might differ from those detained for other reasons, some experiences resonate with findings from a review of qualitative studies including adults. A review found that individuals experienced an unnecessary loss of freedom as they felt other alternatives were available, but also that detention was a sanctuary and way to recover away from life problems [68]. Such views may however change over the course of the detention [53, 68]. A study including young people (16–27 years) found that the majority felt the experience of detention had significant impact on trust to disclose their feelings, which in turn impacted on their post-discharge help seeking. While other participants reported some positive outcomes, those who lacked trust ended up actively withholding how they felt in fear that they would get detained again [69]. Given the impact detention may have on future treatment and a potential cycle of inequality [16], along with calls from international human rights bodies to reduce or eliminate medical coercion [12], more research on children’s and adolescents’ experiences is urgently needed.

Views of parents, caregivers, and care providers are also important. We found no study involving parents’ views and only few that described the experiences of psychiatrists [21, 30, 41, 43]. A major gap remains in the literature on studies exploring how psychiatrists’ perceptions and practices of detaining the youngest patients, their views on addressing inequalities [4], and views on trends or changes in presentations of detained patients in these age groups. In studies on voluntary inpatient treatment parents have reported feeling unprepared, struggling to get access to CAMHS services, and the wider family impact [37,38]. Considering that more older adolescents were detained, it may suggest that parental authority plays a part in the decision to admit a child without their consent. This requires studies to understand how parental views play a part in voluntary or involuntary status of admissions.

While mental health problems among young people are increasing in many countries [70–73], this review could not draw conclusions of trends in detention internationally. Finnish studies showed increases in detention rates [22, 25, 27, 28, 31], while German study showed a decrease in proportion of admissions that were detentions [46]. Changes over time in sub-national samples are less informative, especially as evidence from Finland identified significant variations across regions [29]. Research from Finland has indicated a drastic increase in first treatment of adolescents in psychiatric inpatient care between 1980 and 2010, alongside a decrease in length of stay and global assessment scores [74]. The latter suggests increases in admissions are not related to changes in clinician perceptions of admission thresholds and questions remain regarding higher readmission rates in girls, but possibly due to “the shift from socio-ecological social policies in earlier decades to individual risk and psychopathology-oriented health and social policies” (p.7) [74]. In all age groups, research from England has indicated that reasons for increases in detentions may include the impact from austerity measures, financial crises, and legislative changes [75]. Variation in detention rates between countries is largely unexplained with weak associations between higher incidence of detentions and higher gross domestic product (GDP) and health care expenditure, lower rates of poverty, higher number of inpatient beds, and proportion of foreign born individuals [4]. Kaltiala-Heino [22] also noted that “concern about legal and civil rights of minors may paradoxically increase commitments through more awareness of the obligation to act legally instead of simply deciding over minors without formally recording coercion” (p.57) [22]. In addition, it is difficult to report on the role that diagnoses might play in both the understanding of who gets detained and whether there are differences globally and the appropriateness of detention as there are differences between jurisdictions. The adoption of the ICD-11 in many countries might form a basis to include diagnostic frameworks, wherever appropriate, in recording diagnoses at the point where detention is associated with treatment for a specific condition(s). Changes in detention rates are, however, likely related to a multitude of factors and much of it remains unexplained. Longitudinal nationwide register studies that allow for cross-country comparisons among children and adolescents are lacking and should be a priority for future studies, including comparing detention rates and characteristics with adult populations to better characterise how detention of children and adolescents is used.

Finally, this review has demonstrated the differences in types of detention in studies, which further limits cross-country comparisons. A previous study, which aimed to compare rates of detention and legislative frameworks, highlighted differences in how orders are used and counted in different countries, as no association has been found between detention rates and characteristics of legal criteria [4]. The authors suggested that factors such as detention based on perception of risk vs. urgent need for treatment and coercion within voluntary admissions, where patients are informed they will get detained if they don’t consent to treatment, could impact on detention rates [4]. Differentiating between types of detention, when exploring trends, might be important as in England, for example, there has been a 13% increase in short-term detention over the last five years [76]. The trend in Sect. 5 [2] (assessment up to 72 h) and 5 [4] (nurses’ power to detain) detentions has declining while place of safety orders (s.135 and s.136) have increased in the last two years [76], which may be under circumstances related to lockdowns during Covid-19. Disentangling assessment, treatment, and crisis interventions involving police (in the case of s.135 and s.136 in the UK) might provide information on upstream interventions or resources needed to prevent detention in children and adolescents.

### Limitations

We developed this scoping review with input from a subject librarian to ensure the search strategy was comprehensively designed. However, no protocol was registered prior which is acknowledged as a limitation. This review specifically focused on children and adolescents (aged < 18 years) due to UNCRC’s definition of a child and our interest was also based on the service provision in the UK with specialised child and adolescent mental health services. Within current debates about how adolescence now may span a longer period [18], this restriction may have excluded valuable findings. The review focused on peer reviewed articles, meaning information about trends in detention of children and young people published in statistical reports published by governments or other organisations have been missed, which may give an indication of international trends. As we only reviewed articles published in English, we may have missed information relating to detentions in other contexts than described here.

## Conclusion

Detentions account for about one fifth of psychiatric admissions among children and adolescents, but evidence on trends based on national register data has only been published for a few Western HICs. The circumstances justifying detention and the criteria authorising detention varied between studies, with a mix of clinical factors and observed behaviours. There is some evidence to suggest minority ethnic children and adolescents and those with a history of abuse are disproportionately affected by detention. From a human rights perspective, psychiatric detention based on observed behaviours may be unjustified because of the lack of a ‘true’ mental disorder which benefits from specialist treatment. It may be more justified in emergency situations for urgent and short-term interventions, so understanding what kind of order is being imposed is important. Future research should look in greater detail into different kinds of detention and how they are applied using human rights frameworks. More qualitative studies on the experiences of detention of children and adolescents are urgently needed.

### Electronic supplementary material

Below is the link to the electronic supplementary material.

Supplementary Material 1

Supplementary Material 2

## Data Availability

All related data has been presented within the manuscript.

## Footnotes

1 According to the Norwegian Mental Health Act involuntary treatment can be provided to individuals aged 16 years or older.

2 The study found six arguments for coercive care: (i) protection – people who cannot make their own decisions should be protected by receiving coercive care. This argument is also referred to as ‘weak paternalism’, (ii) solidarity – society has a responsibility towards its citizens and should care for them even if it includes involuntary care, (iii) treatment requirement – the need for treatment is so great, and other avenues have been explored, that coercive care is justified, (iv) clarification – the granting of coercive treatment clarifies to staff, patients and parents that such measures can be used, (v) parent support – if the parental role cannot be fulfilled coercive treatment can be a complement, also when parents are unable or unwilling to take part in the care, (vi) every day care – decision to provide coercive care makes it easier for staff to provide the care the patient needs.

## References

[1] Council of E. European Convention on Human Rights. Strasbourg; 1953.

[2] United Nations Human Rights Office of the High C. International Covenant on Civil and Political Rights. General Assembly resolution 2200A (XXI). 1966.

[3] UN General Assembly. Convention on the Rights of Persons with Disabilities. 2007;A/RES/61/1.

[4] Sheridan Rains L, Zenina T, Dias MC, Jones R, Jeffreys S, Branthonne-Foster S (2019). Variations in patterns of involuntary hospitalisation and in legal frameworks: an international comparative study. The Lancet Psychiatry.

[5] Wessely S, Gilbert S, Hedley M, Neuberger J. Modernising the Mental Health Act: Increasing Choice, Reducing Compulsion 2018 [Available from: https://www.gov.uk/government/publications/modernising-the-mental-health-act-final-report-from-the-independent-review.

[6] Department of Health and Social Care. Draft Mental Health Bill 2022. 2022. Contract No.: CP 699.

[7] Scottish Mental Health Law Review. Scottish Mental Health Law Review 2020 [Available from: https://www.mentalhealthlawreview.scot/.

[8] Keown P, Murphy H, McKenna D, McKinnon I (2018). Changes in the use of the Mental Health Act 1983 in England 1984/85 to 2015/16. Br J Psychiatry.

[9] Rehm J, Shield KD (2019). Global burden of Disease and the impact of Mental and Addictive disorders. Curr Psychiatry Rep.

[10] UNICEF. Convention on the Rights of the Child. 1989.

[11] United Nations Human Rights Office of the High Commissioner. United Nations Convention on the Rights of Persons with Disabilities. 2006.

[12] UN General Assembly. Report of the Special Rapporteur on the right of everyone to the enjoyment of the highest attainable standard of physical and mental health.

[13] UN General Assembly (2017). Report of the Committee on the rights of persons with disabilities.

[14] Walker S, Mackay E, Barnett P, Sheridan Rains L, Leverton M, Dalton-Locke C (2019). Clinical and social factors associated with increased risk for involuntary psychiatric hospitalisation: a systematic review, meta-analysis, and narrative synthesis. The Lancet Psychiatry.

[15] Barnett P, Mackay E, Matthews H, Gate R, Greenwood H, Ariyo K (2019). Ethnic variations in compulsory detention under the Mental Health Act: a systematic review and meta-analysis of international data. The Lancet Psychiatry.

[16] Walker S, Barnett P, Srinivasan R, Abrol E, Johnson S (2021). Clinical and social factors associated with involuntary psychiatric hospitalisation in children and adolescents: a systematic review, meta-analysis, and narrative synthesis. The Lancet Child and Adolescent Health.

[17] Peters MDJ, Marnie C, Tricco AC, Pollock D, Munn Z, Alexander L et al. Updated methodological guidance for the conduct of scoping reviews. JBI Evid Implement. 2021;19(1). 10.1097/XEB.0000000000000277 33570328

[18] Sawyer SM, Azzopardi PS, Wickremarathne D, Patton GC (2018). The age of adolescence. The Lancet Child & Adolescent Health.

[19] Ellila HT, Sourander A, Välimäki M, Warne T, Kaivosoja M (2008). The involuntary treatment of adolescent psychiatric inpatients-A nation-wide survey from Finland. J Adolesc.

[20] Kaltiala-Heino R (2010). Involuntary commitment and detainment in adolescent psychiatric inpatient care. Soc Psychiatry Psychiatr Epidemiol.

[21] Kaltiala-Heino R, Fröjd S (2007). Severe mental disorder as a basic commitment criterion for minors. Int J Law Psychiatry.

[22] Kaltiala-Heino R (2004). Increase in involuntary psychiatric admissions of minors - A register study. Soc Psychiatry Psychiatr Epidemiol.

[23] Khenissi C, Erkolathi R, Ilonen T, Saarijärvi S (2004). Adolescent’s Involuntary Psychiatric Treatment. Psychiatria Fennica.

[24] Kronström K, Ellilä H, Kuosmanen L, Kaljonen A, Sourander A (2016). Changes in the clinical features of child and adolescent psychiatric inpatients: a nationwide time-trend study from Finland. Nord J Psychiatry.

[25] Siponen U, Välimäki M, Kaivosoja M, Marttunen M, Kaltiala-Heino R (2007). Increase in involuntary psychiatric treatment and child welfare placements in Finland 1996–2003: a nationwide register study. Soc Psychiatry Psychiatr Epidemiol.

[26] Siponen U, Välimäki M, Kaivosoja M, Marttunen M, Kaltiala-Heino R (2011). A comparison of two hospital districts with low and high figures in the compulsory care of minors: an ecological study. Soc Psychiatry Psychiatr Epidemiol.

[27] Siponen U, Välimäki M, Kaltiala Heino R (2012). The use of coercive measures in adolescent psychiatric inpatient treatment: a nation-wide register study. Soc Psychiatry Psychiatr Epidemiol.

[28] Sourander A, Turunen MM (1999). Psychiatric hospital care among children and adolescents in Finland: a nationwide register study. Soc Psychiatry Psychiatr Epidemiol.

[29] Sourander A, Korkeila J, Turunen MM (1998). Involuntary psychiatric hospital treatment among 12- to 17-year-olds in Finland: a nationwide register study. Nord J Psychiatry.

[30] Turunen S, Välimäki M, Kaltiala-Heino R (2010). Psychiatrists’ views of compulsory psychiatric care of minors. Int J Law Psychiatry.

[31] Kronström K, Tiiri E, Vuori M, Ellilä H, Kaljonen A, Sourander A. Multi-center nationwide study on pediatric psychiatric inpatients 2000–2018: length of stay, recurrent hospitalization, functioning level, suicidality, Violence and diagnostic profiles. European Child and Adolescent Psychiatry; 2021. 10.1007/s00787-021-01898-0 PMC10147780 34807298

[32] Hanssen-Bauer K, Heyerdahl S, Hatling T, Jensen G, Olstad PM, Stangeland T et al. Admissions to acute adolescent psychiatric units: a prospective study of clinical severity and outcome. Int J Mental Health Syst. 2011;5. 10.1186/1752-4458-5-1 PMC3224249 21211046

[33] Nyttingnes O, Ruud T, Norvoll R, Rugkåsa J, Hanssen-Bauer K. A cross-sectional study of experienced Coercion in adolescent mental health inpatients. BMC Health Serv Res. 2018;18(1). 10.1186/s12913-018-3208-5 PMC5977498 29848338

[34] Clausen L, Larsen JT, Bulik CM, Petersen L (2018). A Danish register-based study on involuntary treatment in Anorexia Nervosa. Int J Eat Disord.

[35] Pelto-Piri V, Kjellin L, Lindvall C, Engström I. Justifications for coercive care in child and adolescent psychiatry, a content analysis of medical documentation in Sweden. BMC Health Serv Res. 2016;16(1). 10.1186/s12913-016-1310-0 PMC4759758 26893126

[36] Ramel B, Täljemark J, Lindgren A, Johansson BA (2015). Overrepresentation of unaccompanied refugee minors in inpatient psychiatric care. SpringerPlus.

[37] Ayton A, Keen C, Lask B (2009). Pros and cons of using the mental health act for severe eating disorders in adolescents. Eur Eat Disorders Rev.

[38] Chaplin R, Roach S, Johnson H, Thompson P (2015). Inpatient Childen and Adolescent Mental Health Services (CAMHS): outcomes of young people with and without intellectual disability. J Intellect Disabil Res.

[39] Corrigall R, Bhugra D (2013). The role of ethnicity and diagnosis in Rates of adolescent psychiatric admission and compulsory detention: a longitudinal case-note study. J R Soc Med.

[40] Eswaravel A, O’Brien A (2018). A retrospective cohort study describing the characteristics of patients under 18 years old in one Sect. 136 suite. Med Sci Law.

[41] Mears A, White R, Lelliott P (2003). Consultant child and adolescent psychiatrists’ knowledge of and attiude to the use of legislation concerning young people with psychiatric disorder. Psychiatr Bull.

[42] Mears A, White R, O’Herlihy A, Worrall A, Banerjee S, Jaffa T (2003). Characteristics of the detained and Informal Child and Adolescent Psychiatric In-Patient populations. Child Adolesc Mental Health.

[43] Mears A, Worral A. A survey of psychiatrists’ views of the Children Act and the Mental Health Act in children and adolescents with mental health problems. Psychiatric hospital care among children and adolescents in Finland: a nationwide register study. 2001;25:304–6.

[44] Nicholls JE, Fernandez CA, Clark AF (1996). Use of mental health legislation in a regional adolescent unit. Psychiatr Bull.

[45] Tolmac J, Hodes M (2004). Ethnic variation among adolescent psychiatric in-patients with psychotic disorders. Br J Psychiatry.

[46] Jendreyschak J, Illes F, Hoffman M, Holtman M, Haas C, Burchard F (2014). Voluntary versus involuntary hospital admission in child and adolescent psychiatry: a German sample. Eur J Child Adolesc Psychiatry.

[47] So P, Wierdsma AI, Kasius MC, Cornelis J, Lommerse M, Vermeiren RRJM (2021). Predictors of voluntary and compulsory admissions after psychiatric emergency consultation in youth. Eur Child Adolesc Psychiatry.

[48] Deolmi M, Turco EC, Pellegrini P, Marchesi C, Pisani F. Psychiatric Emergency in Children and Adolescents: A Retrospective Study in Parma Local Health Unit. Behavioural Neurology. 2021;2021. 10.1155/2021/8848387 PMC8560292 34733375

[49] Voultous P, Tsamadou E, Karakasi MV, Raikos N, Pavlidis P (2020). Involuntar psychiatric hospitalization of children and adolescents in Northern Greece: retrospective epidemiological study and related issues. Psychiatriki.

[50] Christy A, Kutash K, Stiles P (2006). Short term involuntary psychiatric examination of children in Florida. Adm Policy Mental Health Mental Health Serv Res.

[51] Kilgus MD, Pumariega AJ, Cuffe SP (1995). Influence of race on diagnosis in Adolescent Psychiatric inpatients. J Am Acad Child Adolesc Psychiatry.

[52] Lindsey MA, Joe S, Muroff J, Ford BE (2010). Social and clinical factors associated with psychiatric emergency service use and civil commitment among African-American youth. Gen Hosp Psychiatry.

[53] Rice JL, Tan TX, Li Y. In their voices: experiences of adolescents during involuntary psychiatric hospitalization. Child Youth Serv Rev. 2021;126.

[54] Greenham SL, Persi J (2014). The state of Inpatient Psychiatry for Youth in Ontario: results of the ONCAIPS Benchmarking Survey. J Can Acad Child Adolesc Psychiatry.

[55] Persi J, Bird BM, DeRoche C (2016). A comparison of Voluntary and Involuntary Child and adolescent Inpatient Psychiatry admissions. Residential Treat Child Youth.

[56] Smith G, Collings A, Rsw M, Degraaf A. Young people admitted on a form 1 to a general hospital: a worrisome trend. Report No; 2004. p. 7053279189. 10.1093/pch/9.4.228 PMC2720502 19655014

[57] Stein BA, Tanzler L (1988). Morbidity and mortality of Certified Adolescent Psychiatric patients. Can J Psychiatry.

[58] Jaworowski S, Zabow A (1995). Involuntary Psychiatric hospitalization of minors. Med Law.

[59] Geng F, Jiang F, Conrad R, Liu T, Liu Y, Liu H et al. Factors Associated with Involuntary Psychiatric hospitalization of youths in China based on a nationally Representative Sample. Front Psychiatry. 2020;11. 10.3389/fpsyt.2020.607464 PMC7744285 33343433

[60] Park C, McDermott B, Loy J, Dean P. Adolescent admissions to adult psychiatric units: patterns and implications for service provision. Australasian Psychiatry2011. p. 345–9. 10.3109/10398562.2011.601311 21879868

[61] Phillips MR, Chen H, Diesfield K, Cheng HG, Mellsop G, Liu X (2013). China’s New Mental Health Law:reframing Involuntary Treatment. Am J Psychiatry.

[62] World Health O. Mental Health Atlas 2020. Geneva: Organización Mundial de la Salud; 2021. Report No.: 978-92-4-003670-3.

[63] Krendl AC, Pescosolido BA. Countries and Cultural differences in the Stigma of Mental Illness: the East-West divide. J Cross-Cult Psychol.2020(2):149–67.

[64] Erskine HE, Baxter AJ, Patton G, Moffitt TE, Patel V, Whiteford HA (2017). The global coverage of prevalence data for mental disorders in children and adolescents. Epidemiol Psychiatric Sci.

[65] Brazel M, Allison S, Bastiampillai T, Kisely SR, Looi JC (2023). Child and Adolescent Mental Health Services in Australia: a descriptive analysis between 2015-16 and 2019-20. Australas Psychiatry.

[66] Australian Institute for Health and Welfare. Involuntary treatment in mental health care 2023 [Available from: https://www.aihw.gov.au/mental-health/topic-areas/involuntary-treatment#Who-receives.

[67] Lee G, Cohen D (2020). Incidences of Involuntary Psychiatric detentions in 25 U.S. States. Psychiatric Serv.

[68] Seed T, Fox JRE, Berry K (2016). The experience of involuntary detention in acute psychiatric care. A review and synthesis of qualitative studies. Int J Nurs Stud.

[69] Jones N, Gius BK, Shields M, Collings S, Rosen C, Munson M (2021). Investigating the impact of involuntary psychiatric hospitalization on youth and young adult trust and help-seeking in pathways to care. Soc Psychiatry Psychiatr Epidemiol.

[70] Pitchforth J, Fahy K, Ford T, Wolpert M, Viner RM, Hargreaves DS (2019). Mental health and well-being trends among children and young people in the UK, 1995–2014: analysis of repeated cross-sectional national health surveys. Psychol Med.

[71] Patalay P, Gage SH (2019). Changes in millennial adolescent mental health and health-related behaviours over 10 years: a population cohort comparison study. Int J Epidemiol.

[72] Keyes KM, Gary D, O’Malley PM, Hamilton A, Schulenberg J (2019). Recent increases in depressive symptoms among US adolescents: trends from 1991 to 2018. Soc Psychiatry Psychiatr Epidemiol.

[73] Bersia M, Berchialla P, Charrier L, Lemma P, Borraccino A, Nardone P (2022). Mental Well-Being: 2010–2018 Trends among Italian adolescents. Int J Environ Res Public Health 2022.

[74] Holttinen T, Pirkola S, Rimpelä M, Kaltiala R. Factors behind a remarkable increase in adolescent psychiatric inpatient treatment between 1980 and 2010 – a nationwide register study. 10.1080/0803948820211939780. 2021;76(2):120–8. 34185597

[75] Smith S, Gate R, Ariyo K, Saunders R, Taylor C, Bhui K (2020). Reasons behind the rising rate of involuntary admissions under the Mental Health Act (1983): service use and cost impact. Int J Law Psychiatry.

[76] NHS Digital. Mental Health Act Statistics, Annual Figures – 2020-21.

