# Supplementary material for: Detention of children and adolescents under mental health legislation: a scoping review of prevalence, risk factors, and legal frameworks
Source: BMC Pediatr. 2024 Jan 4;24:12. doi: 10.1186/s12887-023-04464-6 (PMC10765764; doi:10.1186/s12887-023-04464-6)
Supplement: Supplementary file 1 — Supplementary Material 1 [file 12887_2023_4464_MOESM1_ESM.docx]

**Supplementary table 2. Overview of legal criteria for detention**

| **Country** | **Reference** | **Legislation** | **Criteria for detention** | **Detention** |
| --- | --- | --- | --- | --- |
| China | [1] | Mental Health Law of China 2012 | High risk of harm to self or others; severe mental disorder as determined by a psychiatrist. In cases of risk to self a legal guardian must agree with the involuntary admission, where risk to others consent from the guardian is not needed [2]. | Not defined. Phillips et al. [16] notes that here is no defined time limit for detentions and no specific times where a clinician needs to re-evaluate the status of the patient. The law stipulates clinicians should re-evaluate when the patient’s clinical status changes and discharge if the criteria are no longer fulfilled. |
| Denmark | [3] | The Danish Mental Health Act (1989) | Psychosis or “similar state of mind”; deterioration of the condition and chances of recovery without treatment; risk to self or others [4]. | The study separated out involuntary admission and detention (a voluntary admission where patient wants to leave and must stay on same reasons as for involuntary admission). Period of admission and detention not specified. |
| Finland | [5-15] | The Finnish Mental Health Act 1990/1116 | Serious mental disorder; condition will deteriorate if not treated; the condition is posing a risk to the individual and/or other people; other types of treatments are not applicable to provide the right intervention; criteria differ to adults in which a narrower criterion of mental illness (which is generally understood to require psychosis) is required rather than severe mental disorder | Siponen et al. [8] defined “The process of involuntary hospitalisation involves assessment by three independent physicians. First a physician refers the minor to a psychiatric hospital with a referral for observation (MI). The patient is placed under observation for a maximum of four days, at the end of which a second physician produces motivated statement of need for involuntary care (MII). The chief psychiatrist in charge then makes the decision on whether the patient is to be detained in involuntary treatment or not (MIII)” (p.662) Siponen et al. [10] defined that “by involuntary treatment, we refer to treatment periods including any stay on an involuntary basis”. At three months the decision to treat involuntarily is reviewed and following a judicial process involuntary treatment can continue for another six months before external review by a physician and the process can be repeated [16]. |
|  | [17, 18] | The Finnish Mental Health Act 1952 (revised 1978); The Finnish Mental Health Act 1991/1116 | No description of criteria in the 1952 Act, only mentions that the criteria were different before 1991. Criteria in the 1991 Act as above | Not defined |
| England and Wales | [19-21] | The Mental Health Act 1983 | Any disorder or disability of the mind; risk to self and others; available appropriate treatment; assessment needed for mental disorder [22]. | Not defined |
|  | [23] | Mental Health Act 1983 | Criteria as above | S.2 (any disorder of disability of the mind which warrants detention in the interests of own health and safety or for protection of others)  S.3 (any disorder or disability of the mind which makes medical treatment in hospital appropriate; necessary for patient's health and safety or protection of others; cannot be provided without detention; and appropriate medical treatment is available)  S.4 (emergency admission, up to 72 hours)  S.37 (hospital order for convicted persons)  S. 38 (interim hospital order)  S.47/S.49 (convicted prisoners removed to hospital). |
|  | [24] | Mental Health Act 1983 | Criteria as above | S.5.2 (detention in hospital for up to 72h)  S.2 (as above)  S.3 (as above) |
|  | [25] | The Mental Health Act 1983 | Criteria as above  Avoiding involuntary treatment for anorexia nervosa was generally accepted and use of the Mental Health Act avoided as until a child turned 18 years their parents could override a decision to refuse treatment. The Mental Capacity Act (2005) amended that so that parents could not override refusal of treatment after the age of 16 years, which is relevant for anorexia nervosa | Not defined |
|  | [26] | The Mental Health Act 1983 | Police officer suspecting an individual suffers from a mental disorder can remove the individual from a public place to keep them in a suitable place of safety until arrangements for assessment and treatment can be arranged | 24 hours in a place of safety (police station, hospital, independent hospital, care home, or residential accommodation provided by social services) (s.136) |
| USA | [27] | The Baker Act (Florida) | Mental illness; risk of harm to self or others; neglect | 72h of involuntary examination |
|  | [28] | Not stated (South Carolina) | Not defined | Not defined |
|  | [29] | Not stated (Philadelphia) | Not defined | Not defined |
|  | [30] | State Statute (south eastern state), chapter 394 | Individuals “deemed by law enforcement, physicians, and/or mental health professionals to be a danger to themselves and/or others due to suicidal/homicidal attempt/ideation, psychosis, and/or extreme aggression” (p.2). | Within this state (Chapter 394 of the state statute), the law mandates involuntary psychiatric hold for individuals who are in the middle of a psychiatric crisis to be three days (i.e., 72 h), during which psychiatric evaluation and treatment are typically provided. After the mandatory stay, further hospitalization can be extended if deemed necessary by the clinical staff. |
| Norway | [31, 32] | Norwegian Mental Health Act (s.2) | Psychotic conditions characterised by severe hyperactivity or violence leading to risk to self or others (risk to life and health); psychotic conditions characterised by severe anxiety or depression leading to risk of suicide attempt or harm to self or others; delirium where detoxification is not a main issue  Mental health conditions of children or adolescents that are not possible to be managed by carers and requires urgent CAMHS input; patients aged 16 years or older can be subject to detention. | Not defined |
| South Israel | [33] | Mental Health Act of 1991 | Immediate physical danger to self or others and mental illness. The two need to be causally linked. | Not defined |
| Germany | [34] | German Civil Law Code (§1631) and PsychKG (federal state laws for psychiatric illnesses) | Civil commitment can be used if the patient is of immediate and psychical danger to self and others, alongside the presence of a mental illness. | Not defined |
| Italy | [35] | Not mentioned | Not defined | Not defined |
| New Zealand | [36] | Mental Health Act | Not defined | Not defined |
| Sweden | [37] | Swedish Compulsory Mental Care Act | "Coercive care may only be given if the patient i) is suffering from a serious mental disturbance, and ii) has an absolute need of inpatient psychiatric care due to his/her mental state and general personal circumstances, and iii) objects to such care. The question of whether the patient, due to mental disturbance, is a danger to others should also be taken into consideration." No difference in criteria for adults and minors. (p.2) | Licensed physician examines the patient and if criteria for coercive care are fulfilled they issue a Care Certificate after which the patient will be taken to a public hospital psychiatric department where the decision is made within 24 hours, following a new examination by a psychiatrist, if coercive care will be needed. Not defined further. |
|  | [38] | Not mentioned | Not defined | Not defined |
| Ontario, Canada | [39, 40] | Mental Health Act Ontario (1990) | Reasons for involuntary referrals includes “a) threat of self-harm and attempt of self-harm and/or likelihood of future self-harm, b) violent behaviour towards another person, causing others to fear for their safety and/or likelihood of future harm, or c) lacking competence to care for the self, and likelihood of suffering serious physical impairment | Form 1 (detention for psychiatric assessment). The form states that the patient is incapable of consenting to treatment and authorises, for a period of seven days, the apprehension of the per-son and detention in a psychiatric facility for a maximum of 72 h. |
|  | [41] | Mental Health Act | As above | Form 1 (detention for psychiatric assessment up to 72h )  Form 3 (involuntary admission for treatment up to 14 days) |
|  | [42] |  | Not described | Not defined |
| Netherlands | [43] | Special Admissions to Psychiatric Hospitals | Conditions for compulsory care in Dutch law includes severity of psychiatric symptoms, danger to self or others, and lack of motivation for treatment | Not defined |
| Greece | [44] | Law 2071/1995 (art.95 and following) | Mental disorder; lacking competence to make decision about health welfare; lack of treatment will lead to deterioration of health; risk to self and/or others (violent actions); other measures have been tried before detention | Not defined |

References

1. Geng, F., et al., *Factors Associated With Involuntary Psychiatric Hospitalization of Youths in China Based on a Nationally Representative Sample.* Frontiers in Psychiatry, 2020. **11**.

2. Phillips, M.R., et al., *China’s New Mental Health Law:Reframing Involuntary Treatment.* American Journal of Psychiatry, 2013. **170**(6): p. 588-591.

3. Clausen, L., et al., *A Danish register-based study on involuntary treatment in anorexia nervosa.* International Journal of Eating Disorders, 2018. **51**(11): p. 1213-1222.

4. Brandt-Christensen, M., *Mental health law in Denmark.* International Psychiatry, 2012. **9**(4): p. 88-90.

5. Kaltiala-Heino, R., *Increase in involuntary psychiatric admissions of minors - A register study.* Social Psychiatry and Psychiatric Epidemiology, 2004. **39**(1): p. 53-59.

6. Kaltiala-Heino, R., *Involuntary commitment and detainment in adolescent psychiatric inpatient care.* Social Psychiatry and Psychiatric Epidemiology, 2010. **45**(8): p. 785-793.

7. Kaltiala-Heino, R. and S. Fröjd, *Severe mental disorder as a basic commitment criterion for minors.* International Journal of Law and Psychiatry, 2007. **30**(1): p. 81-94.

8. Siponen, U., et al., *A comparison of two hospital districts with low and high figures in the compulsory care of minors: An ecological study.* Social Psychiatry and Psychiatric Epidemiology, 2011. **46**(8): p. 661-670.

9. Siponen, U., et al., *Increase in involuntary psychiatric treatment and child welfare placements in Finland 1996-2003: A nationwide register study.* Social Psychiatry and Psychiatric Epidemiology, 2007. **42**(2): p. 146-152.

10. Siponen, U., M. Välimäki, and R. Kaltiala Heino, *The use of coercive measures in adolescent psychiatric inpatient treatment: A nation-wide register study.* Social Psychiatry and Psychiatric Epidemiology, 2012. **47**(9): p. 1401-1408.

11. Turunen, S., M. Välimäki, and R. Kaltiala-Heino, *Psychiatrists' views of compulsory psychiatric care of minors.* International Journal of Law and Psychiatry, 2010. **33**(1): p. 35-42.

12. Kronström, K., et al., *Changes in the clinical features of child and adolescent psychiatric inpatients: a nationwide time-trend study from Finland.* Nordic Journal of Psychiatry, 2016. **70**(6): p. 436-441.

13. Kronström, K., et al., *Multi-center nationwide study on pediatric psychiatric inpatients 2000–2018: length of stay, recurrent hospitalization, functioning level, suicidality, violence and diagnostic profiles.* European Child and Adolescent Psychiatry, 2021.

14. Ellila, H.T., et al., *The involuntary treatment of adolescent psychiatric inpatients-A nation-wide survey from Finland.* Journal of Adolescence, 2008. **31**(3): p. 407-419.

15. Khenissi, C., et al., *Adolescent's Involuntary Psychiatric Treatment.* Psychiatria Fennica, 2004. **35**: p. 131-141.

16. Seppänen, A. and M. Eronen, *Mental health law in Finland.* International psychiatry : bulletin of the Board of International Affairs of the Royal College of Psychiatrists, 2012. **9**(4): p. 91-93.

17. Sourander, A., J. Korkeila, and M.M. Turunen, *Involuntary psychiatric hospital treatment among 12- to 17-year-olds in Finland: A nationwide register study.* Nordic Journal of Psychiatry, 1998. **52**(5): p. 367-371.

18. Sourander, A. and M.M. Turunen, *Psychiatric hospital care among children and adolescents in Finland: a nationwide register study.* Social Psychiatry and Psychiatric Epidemiology, 1999. **34**: p. 105-110.

19. Corrigall, R. and D. Bhugra, *The role of ethnicity and diagnosis in Rates of adolescent psychiatric admission and compulsory detention: A longitudinal case-note study.* Journal of the Royal Society of Medicine, 2013. **106**(5): p. 190-195.

20. Tolmac, J. and M. Hodes, *Ethnic variation among adolescent psychiatric in-patients with psychotic disorders.* British Journal of Psychiatry, 2004. **184**: p. 428-431.

21. Chaplin, R., et al., *Inpatient Childen and Adolescent Mental Health Services (CAMHS): outcomes of young people with and without intellectual disability.* Journal of Intellectual Disability Research, 2015. **59**(11): p. 995-998.

22. Saya, A., et al., *Criteria, procedures, and future prospects of involuntary treatment in psychiatry around the world: A narrative review*, in *Frontiers in Psychiatry*. 2019, Frontiers Media S.A.

23. Mears, A., et al., *Characteristics of the Detained and Informal Child and Adolescent Psychiatric In-Patient Populations.* Child and Adolescent Mental Health, 2003. **8**(3): p. 131-134.

24. Nicholls, J.E., C.A. Fernandez, and A.F. Clark, *Use of mental health legislation in a regional adolescent unit.* Psychiatric Bulletin, 1996. **20**: p. 711-713.

25. Ayton, A., C. Keen, and B. Lask, *Pros and cons of using the mental health act for severe eating disorders in adolescents.* European Eating Disorders Review, 2009. **17**(1): p. 14-23.

26. Eswaravel, A. and A. O’Brien, *A retrospective cohort study describing the characteristics of patients under 18 years old in one section 136 suite.* Medicine, Science and the Law, 2018. **58**(4): p. 222-232.

27. Armitage, C.J., et al., *Completed suicides and self-harm in Malaysia: A systematic review.* General Hospital Psychiatry, 2015. **37**(2): p. 153-165.

28. Kilgus, M.D., A.J. Pumariega, and S.P. Cuffe, *Influence of Race on Diagnosis in Adolescent Psychiatric Inpatients.* Journal of the American Academy of Child and Adolescent Psychiatry, 1995. **34**(1): p. 67-72.

29. Lindsey, M.A., et al., *Social and clinical factors associated with psychiatric emergency service use and civil commitment among African-American youth.* General Hospital Psychiatry, 2010. **32**(3): p. 300-309.

30. Rice, J.L., T.X. Tan, and Y. Li, *In their voices: Experiences of adolescents during involuntary psychiatric hospitalization.* Children and Youth Services Review, 2021. **126**.

31. Hanssen-Bauer, K., et al., *Admissions to acute adolescent psychiatric units: A prospective study of clinical severity and outcome.* International Journal of Mental Health Systems, 2011. **5**.

32. Nyttingnes, O., et al., *A cross-sectional study of experienced coercion in adolescent mental health inpatients.* BMC Health Services Research, 2018. **18**(1).

33. Jaworowski, S. and A. Zabow, *Involuntary Psychiatric Hospitalization of Minors.* Medicine and Law, 1995. **14**: p. 635-640.

34. Jendreyschak, J., et al., *Voluntary versus involuntary hospital admission in child and adolescent psychiatry: a German sample.* European Journal of Child and Adolescent Psychiatry, 2014. **23**: p. 151-161.

35. Deolmi, M., et al., *Psychiatric Emergency in Children and Adolescents: A Retrospective Study in Parma Local Health Unit.* Behavioural Neurology, 2021. **2021**.

36. Park, S., et al., *Seizures in patients with acute pesticide intoxication, with a focus on glufosinate ammonium.* Hum Exp Toxicol, 2018. **37**(4): p. 331-337.

37. Pelto-Piri, V., et al., *Justifications for coercive care in child and adolescent psychiatry, a content analysis of medical documentation in Sweden.* BMC Health Services Research, 2016. **16**(1).

38. Ramel, B., et al., *Overrepresentation of unaccompanied refugee minors in inpatient psychiatric care.* SpringerPlus, 2015. **4**(1): p. 1-6.

39. Persi, J., B.M. Bird, and C. DeRoche, *A Comparison of Voluntary and Involuntary Child and Adolescent Inpatient Psychiatry Admissions.* Residential Treatment for Children and Youth, 2016. **33**(1): p. 69-83.

40. Smith, G., et al., *Young people admitted on a Form 1 to a general hospital: A worrisome trend*, in *Paediatr Child Health*. 2004.

41. Greenham, S.L. and J. Persi, *The State of Inpatient Psychiatry for Youth in Ontario: Results of the ONCAIPS Benchmarking Survey.* J Can Acad Child Adolesc Psychiatry, 2014. **23**(1): p. 1-1.

42. Stein, B.A. and L. Tanzler, *Morbidity and Mortality of Certified Adolescent Psychiatric Patients.* Canadian journal of psychiatry, 1988. **33**: p. 488-493.

43. So, P., et al., *Predictors of voluntary and compulsory admissions after psychiatric emergency consultation in youth.* European Child and Adolescent Psychiatry, 2021. **30**(5): p. 747-756.

44. Voultous, P., et al., *Involuntar psychiatric hospitalization of children and adolescents in Northern Greece: Retrospective epidemiological study and related issues.* Psychiatriki, 2020. **31**: p. 129-139.
