# Supplementary material for: Detention of children and adolescents under mental health legislation: a scoping review of prevalence, risk factors, and legal frameworks
Source: BMC Pediatr. 2024 Jan 4;24:12. doi: 10.1186/s12887-023-04464-6 (PMC10765764; doi:10.1186/s12887-023-04464-6)
Supplement: Supplementary file 2 — Supplementary Material 2 [file 12887_2023_4464_MOESM2_ESM.docx]

**Supplementary table 1. Search strategy**

| **APA PsycInfo** | 1 child.mp.  2 adolescen*.mp.  3 (young people or young person).mp.  4 (minor or minors).mp.  5 infan*.mp.  6 2 or 3 or 4 or 5  7 exp Mental Health Services/ or exp Mental Health/ or exp Mental Disorders/ or psychiatric health care.mp. or exp Psychiatric Hospitals/  8 exp commitment/  9 (detainment or detention).mp.  10 exp Involuntary Treatment/ or exp "Commitment (Psychiatric)"/ or exp Psychiatric Hospitalization/  11 8 or 9 or 10  12 6 and 7 and 11  13 mental health law.mp.  14 mental health legislation.mp.  15 exp Health Care Policy/  16 13 or 14 or 15  17 12 and 16 |
| --- | --- |
| **Embase** | 1 child*.mp.  2 (adolescent or adolescence*).mp. [mp=title, abstract, heading word, drug trade name, original title, device manufacturer, drug manufacturer, device trade name, keyword heading word, floating subheading word, candidate term word]  3 exp adolescent/  4 young people.mp.  5 (minor or minors).mp.  6 1 or 2 or 3 or 4 or 5  7 exp mental health/  8 (mental health or mental illness).mp.  9 (mental disorder or mental illness).mp  10 7 or 8 or 9  11 mental health act.mp.  12 mental health legislation.mp.  13 mental health law.mp.  14 11 or 12 or 13  15 (detainment or detention).mp.  16 involuntary legal status.mp.  17 (involuntary commitment or involuntary hospital).mp.  18 15 or 16 or 17  19 8 and 10 and 18 |
| **Ovid MEDLINE(R)** | 1 child.mp. or Child/  2 Adolescent/ or adolescen*.mp.  3 (young people or young person).mp.  4 (minor or minors).mp.  5 infan*.mp.  6 1 or 2 or 3 or 4 or 5  7 exp mental health services/ or exp mental health/ or exp mental disorders/ or psychiatric health care.mp.  8 "Commitment of Mentally Ill"/ or Involuntary Commitment/  9 (detainment or detention).mp.  10 exp involuntary treatment/ or exp "Commitment mental illness"/ or exp Psychiatric Hospitalization/ or Psychiatric Hospitalisation/  11 8 or 9 or 10  12 6 and 7 and 11  **v** |
